# Possible Protective Effect of *LOXL1* Variant in the Cohort of Chernobyl Catastrophe Clean-Up Workers

**DOI:** 10.3390/genes12081231

**Published:** 2021-08-10

**Authors:** Gabrielė Žukauskaitė, Ingrida Domarkienė, Aušra Matulevičienė, Evelina Marija Vaitėnienė, Justas Arasimavičius, Giedrė Smailytė, Vaidutis Kučinskas, Laima Ambrozaitytė

**Affiliations:** 1 Department of Human and Medical Genetics, Institute of Biomedical Sciences, Faculty of Medicine, Vilnius University, LT-08661 Vilnius, Lithuania; ingrida.domarkiene@mf.vu.lt (I.D.); ausra.matuleviciene@mf.vu.lt (A.M.); evelina.vaiteniene@mf.vu.lt (E.M.V.); justas.arasimavicius@mf.vu.lt (J.A.); vaidutis.kucinskas@mf.vu.lt (V.K.); laima.ambrozaityte@mf.vu.lt (L.A.); 2 Department of Public Health, Institute of Health Sciences, Faculty of Medicine, Vilnius University, LT-03101 Vilnius, Lithuania; giedre.smailyte@nvi.lt; 3 Laboratory of Cancer Epidemiology, National Cancer Institute, LT-08406 Vilnius, Lithuania

**Keywords:** genome-wide association study, protective genome variant, exfoliation syndrome, glaucoma, Chernobyl nuclear disaster, *LOXL1*, Lithuanian population

## Abstract

Ionising radiation (IR) is an environmental factor known to alter genomes and therefore challenge organisms to adapt. Lithuanian clean-up workers of the Chernobyl nuclear disaster (LCWC) experienced high doses of IR, leading to different consequences. This study aims to characterise a unique protective genomic variation in a relatively healthy LCWC group. This variation influenced their individual reaction to IR and potentially protects against certain diseases such as exfoliation syndrome and glaucoma. Clinical and IR dosage data were collected using a questionnaire to characterise the cohort of 93 LCWC. Genome-wide genotyping using Illumina beadchip technology was performed. The control group included 466 unrelated, self-reported healthy individuals of Lithuanian descent. Genotypes were filtered out from the microarray dataset using a catalogue of SNPs. The data were used to perform association, linkage disequilibrium, and epistasis analysis. Phenotype data analysis showed the distribution of the most common disease groups among the LCWC. A genomic variant of statistical significance (Fishers’ exact test, *p =* 0.019), rs3825942, was identified in *LOXL1* (NM_005576.4:c.458G>A). Linkage disequilibrium and epistasis analysis for this variant identified the genes *LHFPL3*, *GALNT6*, *PIH1D1*, *ANKS1B*, and *METRNL* as potentially involved in the etiopathogenesis of exfoliation syndrome and glaucoma, which were not previously associated with the disease. The *LOXL1* variant is mostly considered a risk factor in the development of exfoliation syndrome and glaucoma. The influence of recent positive selection, the phenomenon of allele-flipping, and the fact that only individuals with the homozygous reference allele have glaucoma in the cohort of the LCWC suggest otherwise. The identification of rs3825942 and other potentially protective genomic variants may be useful for further analysis of the genetic architecture and etiopathogenetic mechanisms of other multifactorial diseases.

## 1. Introduction

Natural selection is one of the main evolutionary forces causing species to adapt to their changing environment, as the most advantageous survival traits are selected. The distribution of common genetic variants and fixation of pathogenic ones under the effect of natural selection is crucial for the survival and longevity of a population [1]. In the face of a changing environment and cataclysmic events such as ecological disasters, variants that once were protective may become deleterious. Therefore, ongoing microevolutionary processes lead to transformations in the genetic architecture of adapting populations [2]. To observe the adaptive function of genomic loci and variants, an individual has to be pressured by external and, in some cases, extreme forces. One of the best examples is IR. As a direct effect on the genome, IR creates single or double DNA strand breaks directly or through the production of free radicals and reactive oxygen species and may cause cell death [3].

Genomic studies of individuals who have received high doses of IR are predominantly cytogenetic or analyse the impact of low-dose IR at a genomic sequence level. Therefore, analysis of genomes at the sequence level of individuals who have experienced high doses of IR remains insufficient.

Previous cytogenetic studies that analysed the genomes of LCWC have identified significant changes in genomic stability [4,5]. Dicentric, ring, or fragmented chromosomes are the main aberrations caused by high-level IR exposure. The number of chromosomal changes decreases over time after exposure to IR [6]. However, cells affected by both high and low exposure of IR had higher amounts of cytogenetic aberrations in the group of LCWC even 8 years after the Chernobyl nuclear catastrophe [4,5]. The long-lasting IR effect must be a challenge for genomic and cellular sustainability. Thus, we have to investigate not only chromosomal aberrations resulting as a consequence of IR, but also the genomic capacity for maintaining its own integrity.

About 7000 Lithuanians were sent to work on the clean-up of the catastrophe at the Chernobyl power plant. About half of them are already deceased. A large proportion of clean-up workers have suffered from various illnesses, post-traumatic stress disorder, and depression [7]. High doses of IR may significantly increase the risk for cancer or various tumours or lead to thyroid and eye diseases [8]. However, some of the LCWC have adapted to the long-term effects of IR and are ageing in a relatively healthy manner. This led us to the hypothesis that the reaction to IR may depend on a unique individual genomic variation. This study aims to characterise the unique genomic variation within the LCWC group. Thus, we focused on the association between the phenotypes of the LCWC, the IR dosage they received, and potentially protective genomic variants. For the gene set association analysis in the LCWC group, we created a database (catalogue) of genomic variants that are known to be protective or with their effect status unknown. As a result, we found one association with the *LOXL1* gene variant which is known for its role in exfoliation syndrome (XFS) manifestation and glaucoma. Exfoliation syndrome (MIM#177650) is an age-related systemic disease that affects the extracellular matrix in many ocular tissues. It increases the risk of glaucoma and susceptibility to diseases of elastin-rich connective tissues [9]. *LOXL1* is well known for its contribution to the manifestation of XFS, although no evidence beyond genetic association [10,11,12,13] has been reported, and the genetic architecture of the syndrome remains incompletely understood.

## 2. Materials and Methods

### 2.1. Participants and Samples

The study group consisted of 93 men who worked at the Chernobyl nuclear disaster zone. The study was conducted according to the ethical standards and was approved by the Vilnius Regional Research Ethics Committee (approval No. 2019/4˗1119˗612). Informed consent was obtained from all individuals involved in the study. Venous blood samples were collected in 2019–2020, i.e., about 30 years after the Chernobyl nuclear disaster. DNA was extracted from peripheral blood leukocytes using the phenol-chloroform-isoamyl alcohol method according to laboratory-approved methodology. DNA concentration and purity were determined with a NanoDrop^®^ 1000 spectrophotometer (Thermo Fisher Scientific, Wilmington, DE, USA).

Participants filled out the questionnaire comprising questions about the clean-up process after the Chernobyl nuclear disaster, IR dose, living and working location, and conditions during the clean-up, as well as information on genealogy, lifestyle, and clinical phenotype. The phenotype of the LCWC was evaluated by a clinical geneticist, although all other clinical features were self-reported. In this study, descriptive statistics of IR dosage and clinical phenotype data were calculated using Microsoft Excel. After quality control steps (see Section 2.3), 91 LCWCs were included in further statistical analysis.

The control group consisted of 466 unrelated, self-reported healthy individuals of Lithuanian descent representing the general population (sample collection and high-throughput genotyping was a part of the LITGEN project funded by the European Social Fund under the Global Grant measure, agreement No. VP1-3.1-ŠMM-07-K-01-013). After the quality control steps, the control group for further association analysis was composed of 421 individuals (209 women and 212 men).

### 2.2. Catalogue of Effect Genomic Variants

A catalogue of 144 effect genomic variants from the ClinVar [14] and OMIM [15] databases and scientific publications was compiled. The criteria to include a variant from the databases were (1) clinical significance review status (protective or uncertain) and (2) count of submissions (more than 1). The inclusion criteria for a variant from scientific publications were (1) influence of the variant on gene function (i.e., the variant was expected to alter gene function; mostly loss-of-function) and (2) the frequency of the variant (i.e., rare or previously rare alleles that increased in frequency possibly because of positive effects on the phenotype). Different hereditary conditions were considered as potentially effective. This catalogue (see Supplementary Table S1) was used for the targeted gene-set association analysis.

### 2.3. Genotyping Data Collection and Quality Control

Genome analysis consisted of genotyping, DNA sample quality control, and SNPs quality control steps.

High-throughput genotyping (Illumina HiScanSQ System, Illumina Inc., San Diego, CA, USA) was performed using Illumina Infinium^®^ HTS assay protocol guides (for the genotyping of the general Lithuanian population, Illumina HumanOmniExpress-12 v1.0 and v.1.1. and Infinium OmniExpress-24v1.2 beadchip arrays were used; for the cohort of the LCWC, genotyping was performed using an Infinium OmniExpress-24v1.3 beadchip array kit).

Primary genotyping results were examined and prepared for further analysis using GenomeStudio 2.0 software (https://support.illumina.com/downloads/genomestudio-2-0.html, last accessed on 27 July 2021). Quality parameters for DNA samples were the following [16]: call rate > 97, p10GC > 0.7. To perform SNP quality evaluation, call frequency, GenTrain, and ClusterSep scores were used as quality control parameters [17]. The quality parameters for SNPs were the following: call frequency was 0.97, and the GenTrain value had to be close to 1. SNPs with ClusterSep scores of less than 0.27 were eliminated, and variants in a range between 0.27 and 0.4 were re-clustered manually. Subsequent data quality control and a Hardy–Weinberg equilibrium check were performed using PLINK v1.9 software [18]. Quality control was performed using such features as missingness per individual (0.1), missingness per marker, and Hardy–Weinberg equilibrium (0.001).

After quality control of the genotyping data of the general Lithuanian population was carried out, 421 samples were set for further analysis (45 samples did not reach the cut off value of the call rate parameter or were ruled out due to missing genotypes). After quality control of the genotyping data of the LCWC, all 91 samples were set for further analysis (two were eliminated due to poor genotyping quality). After all of the quality control steps for the SNPs, 700,064 genomic variants (out of the initial 714,238) were set for analysis.

### 2.4. Statistical Analysis

Further analysis was carried out by extracting particular genomic variant genotyping data using the catalogue of effect genomic variants (144 variants in total, see Section 2.2). Filtered variants were tested for Hardy–Weinberg equilibrium using PLINK v1.9 software (https://www.cog-genomics.org/plink/, last accessed on 27 July 2021), and 46 genomic variants passed.

Genotype frequencies were determined in the cohort of the LCWC and compared to the general Lithuanian population (control) group. To rule out population stratification related to the control group’s sex, we performed a χ^2^ test. A χ^2^ or Fisher’s exact test (when the sample size was ≤ 5, α = 0.05) and power calculations for different inheritance models were performed using Rstudio v3.5.2. software (http://www.r-project.com/, last accessed on 21 July 2021). Linkage disequilibrium and epistasis test (α = 0.01) were performed using PLINK v1.9 software.

To interpret the possible molecular outcome, associated genomic variants were analysed using in silico tools and databases: Varsome [19], Uniprot [20], Ensembl [21], ClinVar, OMIM, and GeneMANIA [22].

## 3. Results

### 3.1. Phenotype Analysis

**Figure 1:**
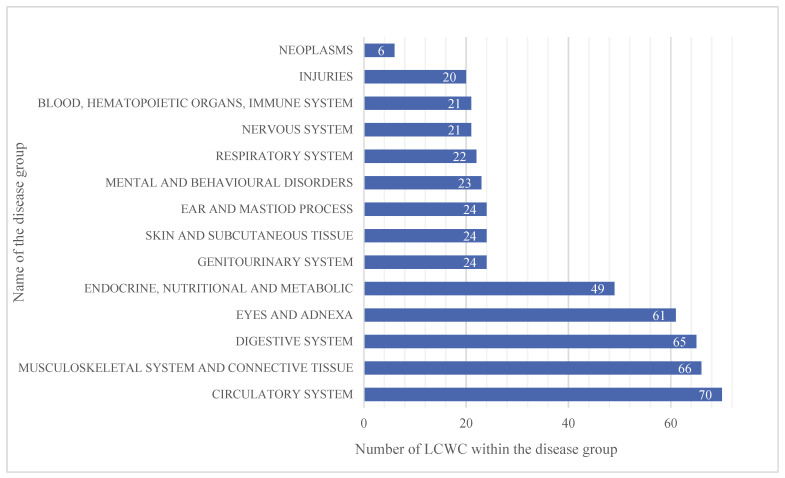
Distribution of the most common disease groups among the LCWC. Diseases of the following groups are the most common in the cohort of Lithuanian clean-up workers: circulatory system; musculoskeletal and connective tissues; digestive system; eyes and adnexa; and endocrine system, nutrition, and metabolism. The *X*-axis depicts the absolute number of LCWC within the disease groups. Some of the participants have diseases from several disease groups. The *Y*-axis depicts disease groups named according to the ICD-10-AM International Statistical Classification of Diseases and Related Health Problems.

Based on the questionnaire of this research study, all the participants were 50–78-year-old men (mean age 64 (SD ± 7)). A majority of the LCWC currently suffer from conditions in these five groups: the circulatory system; musculoskeletal and connective tissues; the digestive system; eye and adnexa problems; and the endocrine system, nutrition, and metabolism. Figure 1 shows the distribution of the disease groups among the LCWC group.

**Figure 2:**
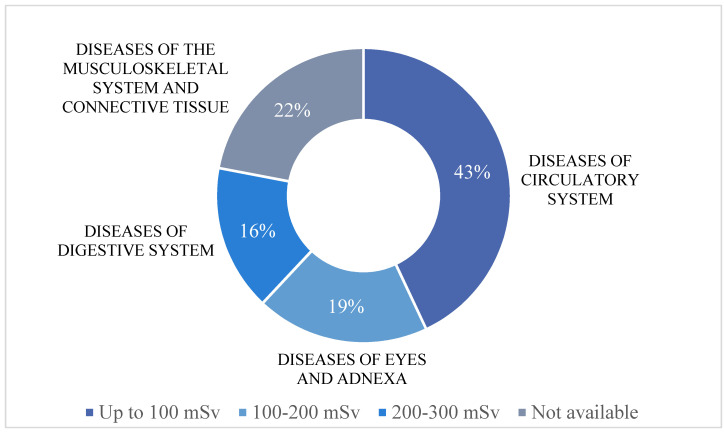
The most common disease groups in the cohort of the LCWC according to IR dose. A significant percentage of participants (43%; 40 of 93 participants) in the cohort of the LCWC experienced IR doses up to 100 mSv. Diseases of the circulatory system predominated in this group. Of the LCWC, 19% (18 of 93 participants) experienced IR doses of 100–200 mSv, with eye and adnexa diseases being the most common in this group. Diseases of the digestive system predominated in the group of the LCWC with IR doses of 200–300 mSv (16%; 15 of 93 participants). In the LCWC group, information about the dose of IR was not available (22%; 20 out of 93 participants), and diseases of the musculoskeletal system and connective tissue were the most common.

We also analysed which common diseases were characteristic of the LCWC based on the IR dose (see Figure 2). No statistically significant differences were found between the incidence of diseases in individuals with different doses of IR.

### 3.2. Gene Set Association Analysis

With the catalogue of effect genomic variants used as a reference, the genotyping data of our sample group were filtered. After targeted association analysis of 46 filtered SNPs, the genomic variant rs3825942 in the gene *LOXL1* (NM_005576.4:c.458G>A; NP_005567.2:p.(Gly153Asp)) was identified. The genotype frequency of the variant among the LCWC differed significantly (see Table 1) from the general Lithuanian population. The Bonferroni correction showed that α = 0.001; therefore, the significance for the rs3825942 genomic variant did not withstand multiple testing analyses. However, this method is the most conservative and increases the rate of false-positive results [23]. Allele frequency analysis showed no statistical significance.

Our study group consisted of men only, and our control group (the general Lithuanian population) was comprised of both men and women to gain extra statistical power. To ascertain that there was no population stratification related to the sex in the control group, we performed a χ^2^ test. It showed no statistically significant difference for the *LOXL1* gene variant in the control group according to sex (χ^2^ = 1.103, *p*-value = 0.576). Statistical power calculations for the *LOXL1* gene variant were performed, taking into account different inheritance models. Analysis showed that power is sufficient (>85%, α = 0.05) for the dominant, additive or 2df models. Although power was insufficient for the recessive model, it was ruled out because *LOXL1* is known to be inherited in an autosomaldominant (MIM#153456) manner.

### 3.3. Linkage Disequilibrium and Epistasis Analysis

To identify genes in linkage disequilibrium with rs3825942 and determine epistasis of the rs3825942 variant, subsequent analyses were performed. The *LOXL1* variant rs3825942 is in linkage disequilibrium and forms a haplotype block with certain other variants (rs2165241, rs1078967, rs4886776, rs8041685, rs8042039, rs4886782, rs2304719, rs750460) in the same gene. Other variants (rs13243476, rs10876168, rs7462) that are also in linkage disequilibrium (r2 value varied from 0.2 to 0.25) are in the genes *LHFPL3*, *GALNT6*, and *PIH1D1*. Analysis of epistasis revealed additional genes such as *ANKS1B* (rs1500733, *p =* 6.5 × 10^−5^, OR = 7.82 (95% confidence interval 2.85–21.46)) and *METRNL* (rs6502043, *p =* 9.8 × 10^−6^, OR = 0.17 (95% confidence interval 0.07–0.41)). These aforementioned genes might be involved in the etiopathogenesis of XFS and glaucoma and were not associated with these conditions previously.

## 4. Discussion

In our study, we characterised the group of the LCWC through analysis of phenotypic and IR dosage data collected using the prepared questionnaire. Lithuanian clean-up workers were dispatched in the areas with the highest radioactive contamination (30 km zone), where they performed various decontamination tasks aimed to control the spread of radioactive pollution (the most toxic activities being the manual collection of granite pieces or decontamination of the power plant itself and equipment that was inside or nearby the power plant) and other activities (for example, driving vehicles to and from the Chernobyl power plant or constructing roads).

According to official data [24], exposure doses in 1986–1989 were up to 100 mSv. This statement supplements our data, which show that a significant percentage (43%) of the LCWC experienced doses up to 100 mSv. The data concerning the amount of IR that workers were exposed to were collected directly from the LCWC and not always supported by documented readings, which are sometimes considered inaccurate [25]. Doses higher than 200 mSv were reported by 16% of the LCWC.

Analysis of phenotype data allowed the distribution of frequencies of common diseases in the cohort of LCWC to be observed. This distribution almost coincides with the data of the general Lithuanian population provided by the Lithuanian Institute of Hygiene [26]. Many of these diseases are age related. Interestingly, only a few of the LCWC have been diagnosed with neoplasms, even though IR is known for its carcinogenic effects and is one of the most common causes of death in the Lithuanian population 45 years of age and older. This paradox may be due to a unique variation in the genomes or the structure of our study group (a majority of LCWC who may have had these diseases might already be deceased). One of the most common disease groups in the cohort of LCWC is eye disease. However, association analysis helped us to identify the genomic variant rs3825942 in the gene *LOXL1* that may be protective against a specific eye disease, exfoliation syndrome.

This gene encodes a LOXL1 enzyme that is required in elastin biogenesis and collagen cross-linking, making it a key element for elastic fibre formation and remodelling [27]. The main roles of LOXL1 include elastin homeostasis and matrix remodelling during injury and the development of fibrosis and cancer [28].

Increased expression of *LOXL1* is observed in fibrotic diseases such as idiopathic pulmonary fibrosis, while decreased expression is reported in XFS [28]. Igo et al. [29] showed that genetic variants in this gene are linked with an increased risk (OR~20) of developing XFS. The associated variant rs3825942 from our study is one of the variants discussed in the Igo et al. [29] study. There are, however, several important points to discuss.

First, according to Butler et al. [30], integrated haplotype scores for common genomic variants and a negative score (–0.453) for the variant rs3825942 indicate a recent positive natural selection, suggesting that the alternative allele might be evolutionarily beneficial and may protect against XSF in the group of LCWC. Furthermore, analysis of genotypes in the LCWC cohort shows a decrease in odds ratios when two of the *LOXL1* gene variant alleles are present (OR(AA) = 0.34 while OR(GA) = 1.84). This points towards a potentially protective effect of the alternative *LOXL1* allele.

Another argument for the protective function of the variant rs3825942 is related to IR. It is known that IR creates free radicals that cause oxidative stress. This stress creates a cellular imbalance of antioxidants (decrease) and pro-oxidants (increase) that causes double-strand DNA breaks [31,32]. Decreased antioxidant levels and the reference allele of the variant rs3825942 are significant factors in the etiopathogenesis of XFS [33,34]. Our study of the LCWC reproduces this finding. During the clean-up of the Chernobyl nuclear catastrophe, the LCWC experienced higher doses of IR than one normally would receive. They thus experienced oxidative stress and a decrease in antioxidants in the body. XFS is a major risk factor for glaucoma, which has been diagnosed only in the LCWC with a homozygous reference allele genotype of the rs3825942 variant. We did not find a diagnosis of XFS in the group of the LCWC. Of the 93 LCWC, three (3.22%) were diagnosed with glaucoma. Concerning the variant rs3825942, LCWC with a homozygous alternative allele and heterozygous genotypes were not found to have glaucoma. This suggests that the alternative allele of rs3825942 may play a role in natural glaucoma prevention and potentially XFS, too. *LOXL1* has also been shown to be upregulated in an oxidative stress environment, which suggests that *LOXL1* has a functional role in cellular stress response and the pathogenesis of XFS [28]. Other studies propose that *LOXL1* gene variants associated with the disease cause decreased gene expression [35] and therefore suggest a protective effect against XFS and glaucoma in an oxidative stress environment.

Finally, rs3825942 variant “risk” alleles might be found reversed in different populations [27,36] in a process known as allele flipping. In this case, the alternative allele is evaluated as a risk allele in some ethnic populations and as protective in others. Such allele frequency variation among populations implies that the variant rs3825942 is not the only one contributing to the disease, although the association is strong [36,37]. Moreover, the variant rs3825942 is present in a large proportion of the general population, the minor allele frequency being 0.2–0.3 according to the dbSNP database. Furthermore, the variant rs3825942 is present in up to 98% of XFS cases and was also found in up to 85% of unaffected individuals [38]. The high frequency of rs3825942 minor alleles and other *LOXL1* gene variants in the general population may suggest that these variants may not be causative in the development of XFS. In addition, we should include environmental factors here as well. Hypoxia, oxidative stress, ultraviolet radiation exposure, and even low folate and high caffeine intake alter *LOXL1* expression [28,39] and likely contribute to XFS.

Other genes are also known for their involvement in XFS [26]. To identify new candidate genes associated with XFS and glaucoma, linkage disequilibrium and epistasis analysis of the *LOXL1* rs3825942 variant was performed. Such analysis may increase the potential possibility to detect new associations and markers of low significance and could explain some missing heritability [40]. In our study, the newly identified linked genes *LHFPL3*, *GALNT6*, *PIH1D1*, *ANKS1B*, and *METRNL* may be involved in the etiopathogenesis of XFS and glaucoma. Using the GeneMANIA gene–gene interaction prediction tool, indirect interactions amongst most of these genes were detected. However, preliminary analysis of gene-gene interactions using the scientific literature showed no biological interactions between these genes. These inconsistent findings suggest that further analysis is needed to examine gene networks and interactions.

## 5. Conclusions

The gene *LOXL1* has important roles in maintaining cell homeostasis. The incidence of XFS and glaucoma is associated with *LOXL1* risk and/or protective genomic variants. In this study, we identified a genomic variant in *LOXL1* (rs3825942; NM_005576.4:c.458G>A) that reached statistical significance in the cohort of Lithuanians who participated in the clean-up of the Chernobyl nuclear disaster compared with the general population of Lithuania. This variant is well known and mainly associated with an increased risk of XFS with glaucoma. However, we propose a different point of view and suggest that it may protect the LCWC against the disease.

The identification of rs3825942 and other potentially protective genomic variants may be useful for the analysis of the genetic architecture of multifactorial diseases such as XFS or glaucoma and supplement knowledge of their etiopathogenesis mechanisms. Although protective genomic variants may reduce the risk of disease, additional genetic mechanisms are involved in this process. The effects of individual genomic variants are important, but the interactions between them, as well as regulatory elements, are also significant. These elements are important for the expression of genomic variants and the onset of the disease. Therefore, further research is needed to investigate not only the diversity of causal genomic variants, but also to deepen our knowledge at the functional level. Functional analysis of rare *LOXL1* gene variants that are conservative in different ethnic groups would be an appropriate next step in the interpretation of XFS disease biology. Transcriptome analysis at the tissue or cell level is also needed to identify gene networks involving *LOXL1* in the pathogenesis of XFS and glaucoma. Once ascertained, the molecular pathways and protective and risk variants could be used as therapeutic targets.

## Figures and Tables

**Table 1:** Summary statistics of the variant rs3825942 in the gene *LOXL1*, which showed statistical significance.

Study Group	AA Genotype Count	GA Genotype Count	GG Genotype Count	Minor Allele Frequency	Fisher’s Exact Test, *p*-Value	Odds Ratio (AA Genotype)	Odds Ratio (GA Genotype)
Cases	1	35	55	0.203	*p* = 0.019	0.34 (95% CI 0.04–2.63)	1.84 (95% CI 1.14–2.95)
Controls	16	104	301	0.162			

## Data Availability

All data generated or analysed during this study are included in this published article and its supplementary information files. Additional data may be available upon request.

## Supplementary Materials

The following are available online at https://www.mdpi.com/article/10.3390/genes12081231/s1, Supplementary Table S1: Catalogue of effect genomic variants.
